# Simultaneous mutation detection of three homoeologous genes in wheat by High Resolution Melting analysis and Mutation Surveyor^®^

**DOI:** 10.1186/1471-2229-9-143

**Published:** 2009-12-04

**Authors:** Chongmei Dong, Kate Vincent, Peter Sharp

**Affiliations:** 1 Plant Breeding Institute, University of Sydney, PMB 4011, Narellan NSW 2567, Australia; 2 Australian Centre for Plant Functional Genomics, PMB 1, Glen Osmond SA 5064, Australia

## Abstract

**Background:**

TILLING (Targeting Induced Local Lesions IN Genomes) is a powerful tool for reverse genetics, combining traditional chemical mutagenesis with high-throughput PCR-based mutation detection to discover induced mutations that alter protein function. The most popular mutation detection method for TILLING is a mismatch cleavage assay using the endonuclease CelI. For this method, locus-specific PCR is essential. Most wheat genes are present as three similar sequences with high homology in exons and low homology in introns. Locus-specific primers can usually be designed in introns. However, it is sometimes difficult to design locus-specific PCR primers in a conserved region with high homology among the three homoeologous genes, or in a gene lacking introns, or if information on introns is not available. Here we describe a mutation detection method which combines High Resolution Melting (HRM) analysis of mixed PCR amplicons containing three homoeologous gene fragments and sequence analysis using Mutation Surveyor^® ^software, aimed at simultaneous detection of mutations in three homoeologous genes.

**Results:**

We demonstrate that High Resolution Melting (HRM) analysis can be used in mutation scans in mixed PCR amplicons containing three homoeologous gene fragments. Combining HRM scanning with sequence analysis using Mutation Surveyor^® ^is sensitive enough to detect a single nucleotide mutation in the heterozygous state in a mixed PCR amplicon containing three homoeoloci. The method was tested and validated in an EMS (ethylmethane sulfonate)-treated wheat TILLING population, screening mutations in the carboxyl terminal domain of the *Starch Synthase II *(*SSII*) gene. Selected identified mutations of interest can be further analysed by cloning to confirm the mutation and determine the genomic origin of the mutation.

**Conclusion:**

Polyploidy is common in plants. Conserved regions of a gene often represent functional domains and have high sequence similarity between homoeologous loci. The method described here is a useful alternative to locus-specific based methods for screening mutations in conserved functional domains of homoeologous genes. This method can also be used for SNP (single nucleotide polymorphism) marker development and eco-TILLING in polyploid species.

## Background

Detection of SNPs in genes of interest, whether induced or endogenous, is a powerful tool to explore gene function and to identify desired mutations for breeding. TILLING has proven to be a valuable methodology for reverse genetics, combining traditional chemical mutagenesis with high-throughput PCR-based mutation detection. As a post-genomics tool, TILLING is not only useful for functional genomics [1], but is also effective for crop improvement [2]. TILLING produces a large chemically mutagenized population with random mutations across the genome, so that an efficient mutation detection method is essential. SNP discovery methods used in TILLING include full sequencing [3], denaturing high-pressure liquid chromatography (dHPLC) [4] and heteroduplex mismatch cleavage assay using endonuclease CelI followed by sequencing [5]. Among these, the mismatch cleavage assay has high sensitivity in pooled samples, and is therefore high-throughput and low cost. Other mutation scanning methods such as single-strand conformational polymorphism (SSCP) [6], denaturing gradient gel electrophoresis (DGGE) [7] and technologies such as pyrosequencing [8] and mass spectrometry (MS) [9] have advantages and disadvantages regarding sensitivity, throughput, cost and simplicity. Heteroduplex mismatch cleavage assay works in any PCR amplicon (usually 0.5-1.5 kb) and any sequence context. The only requirement for heteroduplex assay is the purity of a PCR product. Therefore, PCR reactions for heteroduplex assay are performed using gene-specific primers at high stringency. However, these conditions are sometime difficult to achieve when TILLING a polyploid species. For TILLING in soybean, a recent allotetraploid species [10], a restriction enzyme digestion of the genomic DNA before PCR was added to the method in an attempt to reduce the homoeologous complexity [11], but this method would not work without a locus-specific restriction site.

Bread wheat (*Triticum aestivum*) is an allohexaploid species with three closely related genomes. Most wheat genes are present as three similar sequences of homoeologous loci with high exonic homology and lower homology in introns. Locus-specific primers can usually be designed in intron regions, as shown in wheat waxy genes [2]. However, some wheat genes have high homology among the three homoeologous loci even in introns, so that locus-specific PCR is not easily achievable. Here we report a new method using High Resolution Melting (HRM) analysis and Mutation Surveyor^® ^to screen mutations in the carboxyl terminal domain of the *Starch Synthase II *(*SSII*) gene allowing simultaneous screening of the three homoeologous loci. Conserved regions of a gene which can be identified from multiple sequence alignment of a large number of divergent orthologous genes are believed to have high functional significance http://pfam.sanger.ac.uk/. Mutations in these conserved sequences will have a high likelihood of being deleterious, which is often the purpose of TILLING. For effectively screening mutations in the conserved regions, where locus-specific primers are not easy to obtain, we developed this method allowing simultaneous mutation detection in a functional domain of all three homoeologous genes in hexaploid wheat.

HRM analysis is an extension of previous DNA melting (dissociation) analysis enabled by the new generation of fluorescent dsDNA dyes [12]. These dyes, such as LCGreen and CYTO^®^9, have low toxicity to PCR and can therefore be used at high concentration to saturate the dsDNA PCR product. Greater dye saturation means there is less dynamic dye redistribution to non-denatured regions of nucleic strands during melting so that the measured fluorescent signals have higher fidelity [12,13]. The combination of these characteristics provides greater melt sensitivity and higher resolution melt profiles making it possible to detect SNPs in PCR amplicons, even in somatic mutations and methylations [14-18]. Mutation Surveyor^® ^(SoftGenetics, State College, PA, USA) is a commercially available software for DNA variation analysis that allows automatic mutation detection in sequence traces. Mutation Surveyor^® ^is claimed to detect > 99% of mutations, with sensitivity to the mutant allele extending down to 5% of the primary peak (mosaic or somatic mutations) provided the sequence quality meets a minimum Phred score of 20. The method presented here was tested and validated in an EMS (ethylmethane sulfonate)-treated wheat TILLING population [19], targeting the *SSII *genes.

## Rsults

### Mutation Surveyor^® ^can detect heterozygous mutations in an ampilcon containing three homoeoloci

**Figure 1:**
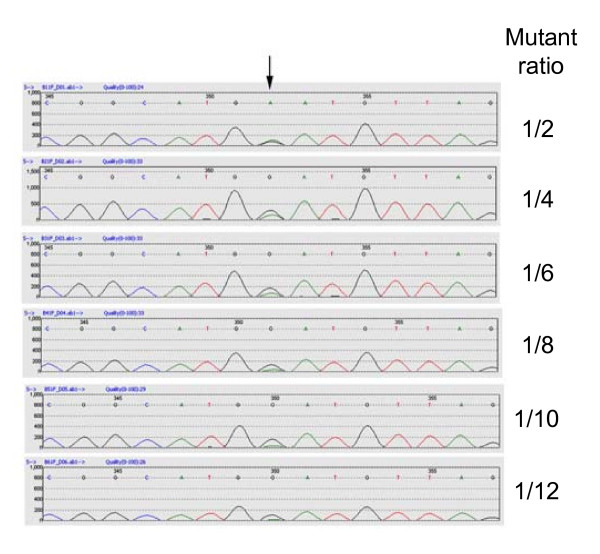
**Mutation Surveyor^® ^software detects a mutant allele in pooled DNA**. Sequence traces (forward traces) from the Graphical Analysis Display of Mutation Surveyor^® ^are shown. The arrow indicates a G to A mutation detected by Mutation Surveyor^® ^in mutant/non-mutant mixed samples with the fraction of mutant allele in pooled DNA being 1/2, 1/4, 1/6, 1/8, 1/10 and 1/12.

Chemically treated TILLING populations contain single-nucleotide changes in the genome. To detect such induced mutations in a PCR reaction end-point containing fragments of three homoeoloci in wheat means the software should be sensitive enough to detect a 1:5 ratio of mutant:background signal in the case of a heterozygous mutation. To test the software sensitivity, a previously identified heterozygous mutant (G1642A in *Wx-D1*) was used to mix with non-mutant DNA to form mutant:non-mutant ratios of 1:0, 1:1, 1:2, 1:3, 1:4 and 1:5 so that the mutant allele fractions in the pooled DNA were 1/2, 1/4, 1/6, 1/8, 1/10 and 1/12. These six samples were used to amplify the Wx7D3 fragment [2] and sequenced in both directions. The sequence data were analyzed with Mutation Surveyor^® ^software set to check bi-directional (2D) small peaks. Due to the nature of sequencing, artefact peaks may appear as real data. However, artefact sequencing peaks rarely occur at the same position in both forward and reverse directions. Using the 2D setting of the software increases the sensitivity and accuracy. Figure 1 shows that the software is able to detect the known mutation in up to a 1/10 dilution. Table 1 shows the Mutation Surveyor^® ^report indicating the mutation position and score. The mutation score is used by the software to call a mutation and rank its confidence level. It is a measure of the probability of error and is based on the ratios of noise level, the overlapping factor and the dropping factor used by the software. The first two samples (1/2 and 1/4 mutant allele) had mutation scores from 9 to 43; other samples had a score of 7 (Table 1). These scores may be used as an indication of the possible zygosity status of a mutant. Due to the nature of sequencing, however, peak heights may be quite variable so it is important that both directions are examined when the mutation score is used as an indication of the zygosity. To test if the software is able to detect a heterozygous mutation in an amplicon containing three homoeoloci of wheat, a *SSII *gene fragment was screened for SNP mutations in a TILLING population.

**Table 1:** The mutation report of Mutation Surveyor^® ^after sequence trace analysis of mutant/non-mutant mixed samples.

Mutant allele in pooled DNA	Sample File	Reference File	Direction	Mutation *	Score
1/2	B11F_D01.ab1	Q7D3_F_G08.ab1	Forward	(352)G>GA$20	20
1/2	B11R_C01.ab1	Q7D3_R_G09.ab1	Reverse	(392)G>GA$43	43

1/4	B21F_D02.ab1	Q7D3_F_G08.ab1	Forward	(352)G>GA$9	9
1/4	B21R_C02.ab1	Q7D3_R_G09.ab1	Reverse	(392)G>GA$16	16

1/6	B31F_D03.ab1	Q7D3_F_G08.ab1	Forward	(352)G>AG$7	7
1/6	B31R_C03.ab1	Q7D3_R_G09.ab1	Reverse	(392)G>AG$7	7

1/8	B41F_D04.ab1	Q7D3_F_G08.ab1	Forward	(352)G>AG$7	7
1/8	B41R_C04.ab1	Q7D3_R_G09.ab1	Reverse	(392)G>AG$7	7

1/10	B51F_D05.ab1	Q7D3_F_G08.ab1	Forward	(352)G>AG$7	7
1/10	B51R_C05.ab1	Q7D3_R_G09.ab1	Reverse	(392)G>AG$7	7

1/12	B61F_D06.ab1	Q7D3_F_G08.ab1	Forward	n.a.	n.a.
1/12	B61R_C06.ab1	Q7D3_R_G09.ab1	Reverse	n.a.	n.a.

*Mutation report indicates the position (in brackets), the base change (G>GA) and the score (the number after the $ sign).

**Figure 2:**
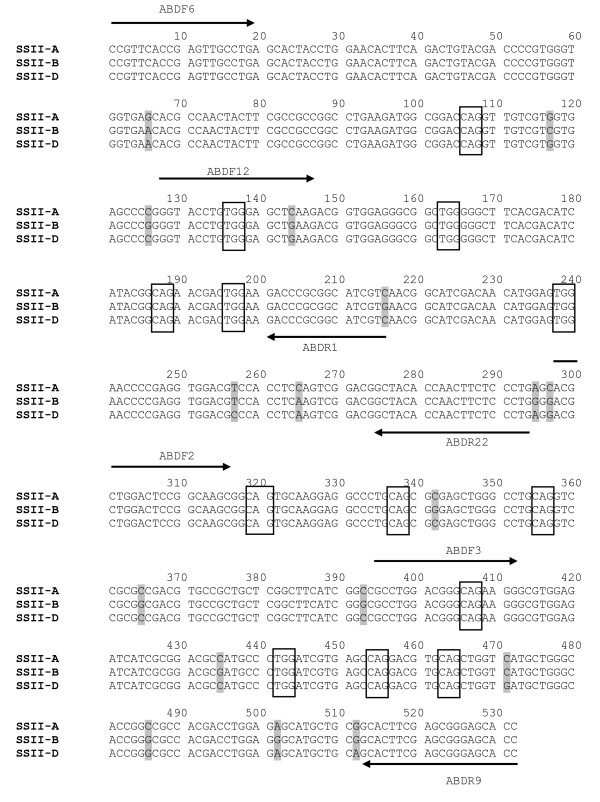
**An alignment of three homoeologous sequences of *SSII***. A gene fragment of *SSII *from primer ABDF6 to ABDR9 is aligned to show three homoeologous loci with 17 SNPs (gray highlighting). The codons (CAG and TGG) which can mutate to premature stop codons are indicated in boxes. Arrows indicate the positions of primers designed for PCR and HRM analysis. The four primer pairs are: ABDF6 and ABDR1, ABDF12 and ABDR22, ABDF2 and ABDR9, and ABDF3 and ABDR9.

**Figure 3:**
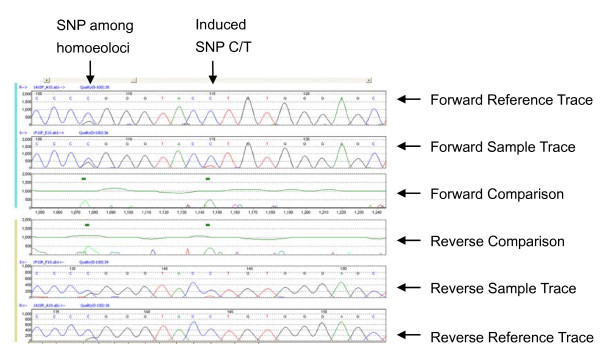
**Mutation Surveyor^® ^detects single nucleotide changes**. An example of Graphic Analysis Display showing that Mutation Surveyor^® ^detects single nucleotide changes in an amplicom containing three homoeologous *SSII *fragments.

The wheat *SSII *genes/homoeoloci (GenBank accessions AB201445, AB201446 and AB201447) are each approximately 7 kb, have eight exons, and share more than 96% identity [20]. By analysis of the gene sequence with CODDLE (for Codons Optimized to Detect Deleterious Lesions; http://www.proweb.org/coddle/), we identify that the last exon contains catalytic domains. This carboxyl terminal is long (957 bp) and very conserved among the three homoeoloci. It was chosen for mutation detection in this study due to the high probability that missense mutations in this exon will have deleterious effects on the enzyme activity, and it has a large number of TGG and CAG codons that can mutate to premature stop codons (Figure 2). The partial exon was PCR amplified using primers ABDF6 and ABDR9 (Figure 2) in 192 TILLING lines. PCR products were purified and sequenced in both directions, and then analyzed by Mutation Surveyor^® ^Software. The initial analysis identified 26 mutants (Additional file 1). An example of a mutant sequence trace analyzed by the software is shown in Figure 3. If these 26 mutants in this 532 bp fragment are all true mutants, then the mutation frequency (26/532 × 3 × 192 bp) was about 1 in 12 kb, which is very high compared to the frequency of about 1 in 24 kb from the screening of waxy gene [19] and other genes (unpublished data) in the same population. It is possible that some false positives are included in this initial analysis. These 26 mutations were re-examined with Mutation Surveyor^®^, and the mutation call thresholds were set to accept the mutation when the mutation height is near or above 500 and the background noise in surrounding base pairs is zero. With these more stringent criteria, some of the mutants were identified as possible false positive mutants. In the following HRM analysis, some were confirmed as false positives. Table 2 lists the mutants identified and confirmed by HRM analysis. Among these 17 mutants, five had a mutation score equal or greater than 10, indicating a possible homozygous mutation. Others had scores of seven, possibly heterozygotes. The apparent percentage of homozygotes (29.4%) is similar to previous findings [19].

**Table 2:** 17 mutations are identified in 192 TILLING lines in ABD6-9 after Mutation Surveyor^® ^analysis of sequence traces and confirmation by HRM analysis.

No	Sample	Mutation Surveyor report	Position in ABD6-9	Position in Gene (SSII-A)	Codon change	Amino acid change	Mutation type
1	3D7	(16)C>CT$7*	C34T	C5999T	cac/tac	H501Y	missense

2	1D3	(25)C>CT$10	C43T	C6008T	ctg/ttg	L504L	silent

3	3F10	(65)C>CT$7	C83T	C6048T	gcc/gtc	A517V	missense

4	1C8	(85)G>AG$7	G103A	G6068A	gac/aac	D524N	missense

5	1F10	(115)C>CT$7	C133T	C6098T	ctg/ttg	L534L	silent

6	1D8	(129)G>AG$7	G147A	G6112A	aag/aaa	K538K	silent

7	3E5	(129)G>AG$7	G147A	G6112A	aag/aaa	K538K	silent

8	3A8	(148)G>AG$7	G166A	G6131A	ggg/agg	G545R	missense

9	1D9	(151)C>CT$28	C169T	C6134T	ctt/ttt	L546F	missense

10	3A4	(159)C>CT$11	C177T	C6142T	gac/gat	D548D	silent

11	3H9	(187)C>CT$7	C205T	C6170T	cgc/tgc	R558C	missense

12	1D5	(300)G>AG$7	G318A	G6283A	cgg/cga	R595R	silent

13	3D8	(306)C>CT$7	C324T	C6289T	tgc/tgt	C597C	silent

14	3F6	(342)C>CT$7	C360T	C6325T	gtc/gtt	V609V	silent

15	1B2	(361)C>CT$13	C379T	C6344T	ctc/ttc	L616F	missense

16	3B7	(365)G>AG$7	G383A	G6348A	ggc/gac	G617D	missense

17	1E4	(387)G>AG$7	G405A	G6370A	ggg/gga	G624G	silent

*The reverse trace of this mutation had a score of 46.

### High Resolution Melting (HRM) analysis of the *SSII *mutants

**Figure 4:**
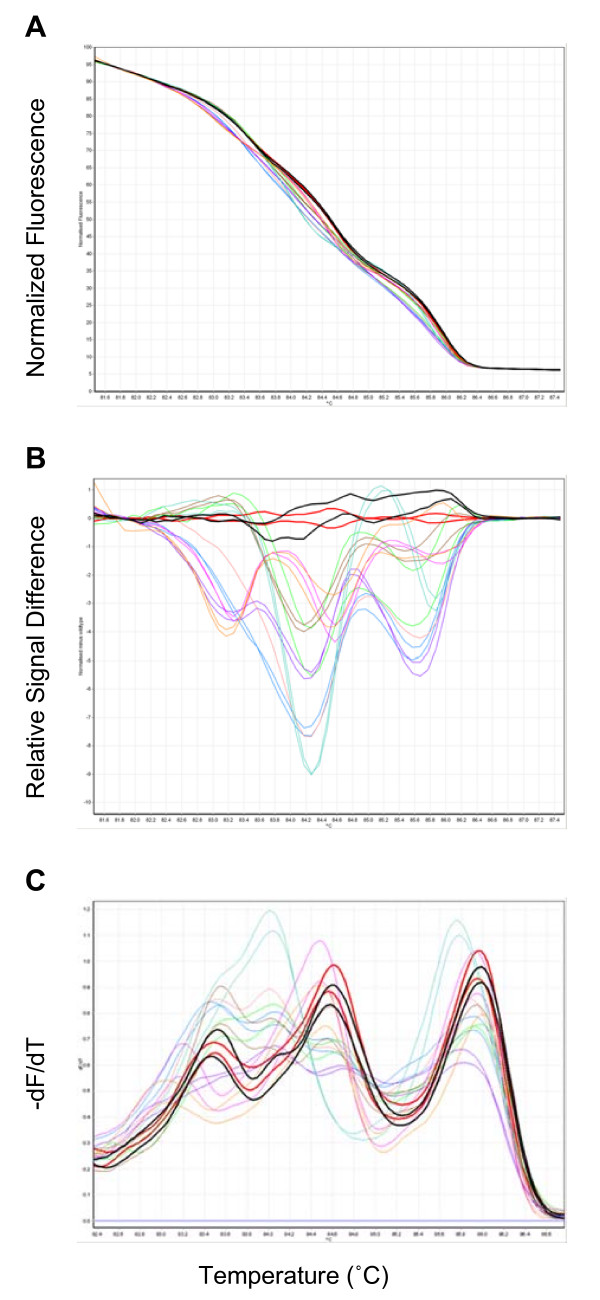
**Amplicon melting analysis of fragment ABD6-1**. Amplicon melting analysis of fragment ABD6-1 in duplicated non-mutant and mutant samples, showing the normalized melting curve (A), difference plot (B) and derivative melting curve (C). Non-mutants are shown in red and black (thick lines). Mutants are (as in Table 2) No3 C83T (blue), No4 G103A (green), No5 C133T (salmon, one PCR did not work, only one sample shown), No6 G147A (brown), No7 G147A (magenta), No8 G166A (purple), No9 C169T (aqua) and No10 C177T (orange).

To test the sensitivity of HRM in scanning for SNPs in mixed PCR fragments, a number of primer pairs were designed to have amplicon sizes between 100 to 250 bp in the ABD6-9 fragment. Three primer pairs were chosen due to their good amplification levels and distinctive melting peaks in the derivative plot; ABDF6 and ABDR1 for amplicon ABD6-1, ABDF12 and ABDR22 for amplicon ABD12-22, and ABDF2 and ABDR9 for amplicon ABD2-9 (Figure 2). Mutants No3 to No10 (Table 2) along with two non-mutant samples were analysed by HRM using ABDF6 and ABDR1 as primers. Each reaction was duplicated. Figure 4 shows the normalized melting curve, difference plot and derivative melting curve of ABD6-1. In the derivative melting curve (Figure 4C), three melting peaks were detected in non-mutants, indicating dynamic melting behavior of the ABD6-1 fragment, possibly due to its high GC content, secondary structures and intrinsic SNPs among the three loci. Despite the complex melting behavior, all mutants tested had shifts in melting peaks from that of the non-mutant. The normalized melting curve (Figure 4A) and the difference plot (Figure 4B) also show that the melting curve shape and the signal difference of the mutants was distinctive from those of the non-mutant. HRM analysis is able to detect mutations in mixed PCR fragments containing other SNPs (among the homoeologous loci). The high sensitivity of HRM to detect SNPs in a complex genome such as wheat should allow the use of this method for scanning mutations in a TILLING population before sequencing. Amplicons ABD12-22 and ABD2-9 were also analyzed by HRM using mutants listed in Table 2 and Additional file 1. Both amplicons are suitable for HRM analysis and mutants had peaks shifted towards a lower temperature (Additional files 2 and 3). It is known that a change from C to T, or G to A will lower melting temperature.

### Detecting unknown mutations using HRM and Mutation Surveyor^® ^analysis

**Figure 5:**
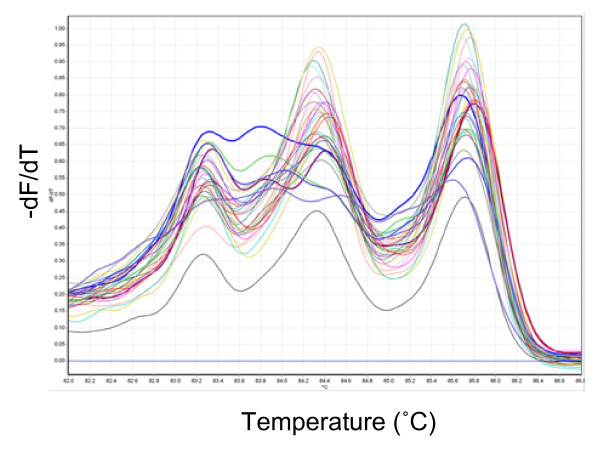
**Amplicon melting analysis of fragment ABD6-1 in 32 blind samples**. Amplicon melting analysis of fragment ABD6-1 in 32 blind samples, showing five samples with altered melting behavior (thick lines) compared to other samples.

Discovering unknown mutations is a more challenging task than determining the presence of known lesions. To test if HRM is sensitive enough to detect rare unknown mutations in a large population in which most samples are non-mutant, 32 samples were random chosen from the 192 samples previously sequenced, and HRM analysed in a blind fashion with amplicon ABD6-1. In this assay, five samples with abnormal melting were discovered (Figure 5) and sequence analyses showed they were mutants. Another three samples had small differences in melting behavior compared to that of non-mutant, but they were not mutants as determined by sequence analyses. Other samples with normal melting were confirmed by sequence data as non-mutant. Therefore, 100% of the mutations were detected.

For TILLING, a large population is needed for finding useful mutants, so the mutation scanning method has to be high-throughput. To use HRM analysis in a high-throughput fashion, an assay to detect mutations in amplicons ABD6-1, ABD12-22 and ABD2-9 in 140 blind unknown samples was conducted. At the same time, fragment ABD6-9 of these 140 samples were sequenced, and the sequence traces were analysed with Mutation Surveyor^® ^using stringent criteria. Results of the two independent assays are compared in Table 3. From HRM analysis of ABD6-1, 15 samples with aberrant melting were identified. Sequence analysis of these 140 samples with Mutation Surveyor^® ^identified eight mutants in the ABD6-1 region with seven detected by HRM analysis and one not detected by HRM of ABD6-1, but detected by HRM of ABD12-22. HRM on fragment ABD12-22 had better sensitivity (100%, Table 3) in detecting unknown mutations compared to ABD6-1 and ABD2-9, assuming that Mutation Surveyor^® ^analyses are 100% correct. All three fragments had some false positives in HRM analysis, ranging from 2.8% to 7.1%.

**Table 3:** Comparison of results from independent HRM and Mutation Surveyor^® ^analysisof 140 TILLING lines.

Fragment	HRM Scanning			Mutation Surveyor^®^			HRM Sensitivity^4^	
	
	Mut^1^	T^2^	F^3^	Mut^1^	HRM detected	HRM un-detected	% sensitivity^4^	% false positive rate^4^
ABD6-1	15	7	8	8	7	1	87.5	5.7
ABD12-22	10	6	4	6	6	0	100	2.8
ABD2-9	15	5	10	6	5	1	83.3	7.1

^1^Number of mutations identified by HRM or Mutation Surveyor^®^.

^2^T = True mutants that sequences contain mutations confirmed by Mutation Surveyor^®^.

^3^F = False mutants that sequences do not contain mutations confirmed by Mutation Surveyor^®^.

^4^%sensitivity = true positive/(true positive + false negative); %false positive rate = false positive/total number of sample analysed; assuming Mutation Surveyor^® ^analyses are 100% correct.

### Progeny testing and cloning

**Figure 6:**
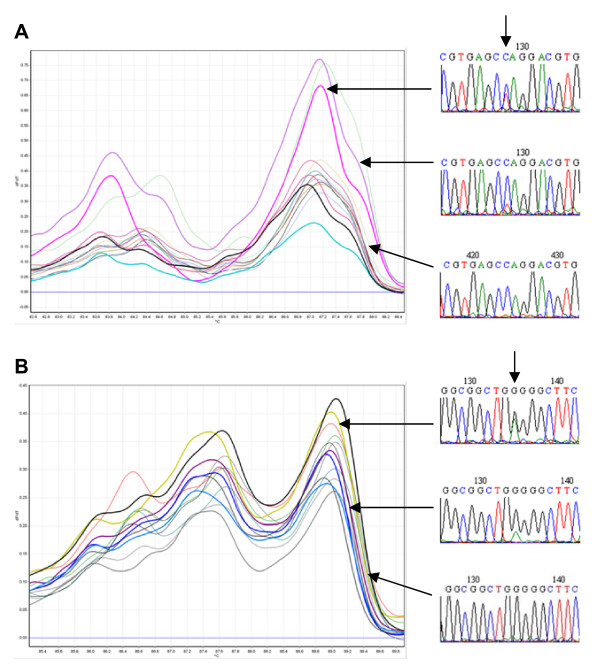
**Progeny tests of mutants 4A7 and 4D7**. Progeny tests of mutants 4A7 (C454T, Q641*) and 4D7 (G165A, W544*). (A) Twelve segregating M2 seedlings of 4A7 were analysed by HRM in ampilcon ABD3-9, four samples showed mutant-like melting peaks (thick lines). The thick black line is the known mutant control. (B) Ten segregating M2 seedlings of 4D7 were analysed by HRM in amplicon ABD6-1, seven samples showed mutant-like melting peaks (thick lines). The thick black line is the known mutant control. Representative sequence traces are shown on the right; homozygote is at the top, heterozygote in the middle and non-mutant at the bottom. Vertical arrows show the mutation positions.

From 140 samples screened in the ABD6-9 fragment by HRM and sequencing, two mutants were found to have nonsense mutations, one was 4A7 (C454T, Q641*) and the other, 4D7 (G165A, W544*). Segregating M2 seeds of these two lines were used in a progeny test. Twelve M2 seedlings of 4A7 and 10 M2 seedlings of 4D7 were analyzed by HRM and sequencing. The mutation in 4A7 is located near the 3'-end of fragment ABD2-9. To increase the sensitivity of HRM, fragment ABD3-9 was chosen for HRM analysis. Figure 6A shows four samples with mutant-like melting peaks. These four samples and one chosen from non-mutant-like samples were sequenced and it was revealed that the four samples were all mutants; one being a possible homozygous mutant and other three were heterozygous. The one showing non-mutant behavior of melting was confirmed by sequence as non-mutant. Figure 6B shows the ABD6-1 melting analysis of 4D7 progenies. Seven mutant-like curves were identified. Sequence analysis confirmed two of the seven were homozygous mutants and other five were heterozygous. Two samples with non-mutant-like melting curves were confirmed by sequence as non-mutant. Homozygous mutants were determined by comparing the ratio of two overlapping peaks with that of neighboring SNPs (intrinsic SNPs among three loci) and a mutation score greater than seven as reported by Mutation Surveyor^®^.

PCR products ABD3-9 (for 4A7) and ABD6-1 (for 4D7) amplified from homozygous progeny of 4A7 and 4D7 were cloned with the pGEM^®^-T Easy vector. From eight sequenced clones of 4A7, two had the mutation and the sequence belonged to the A genome. The other six clones were either B genome or D genome lacking the mutation. From seven sequenced clones of 4D7, one had the mutation which was in the A genome. The other six clones were either B genome or D genome lacking the mutation. The locations of both mutations were therefore identified.

## Discussion

TILLING is a reverse genetics tool for studying gene function. The most desirable mutations in TILLING are those causing complete or partial inactivation of the targeted gene product. Screening mutations in a conserved region or functional domain will increase the efficiency and speed for finding such deleterious mutants. The method described in this report is suitable for screening a functional domain of a gene in a polyploid species such as wheat. In plants, polyploidy is very common and many crops are polyploid, e.g. wheat, oats, potato, cotton [10]. TILLING in polyploids, especially autopolyploids can cause complications in mismatch cleavage assays [11]. HRM scanning can be an alternative choice. Although amplicons for HRM analysis are shorter than that used in mismatch cleavage assay, HRM is a closed-tube, low cost and fast assay; no digestion and gel separation steps are required.

The bread wheat *SSII *gene is very conserved among three homoeoloci, especially within the C-terminal domain. The method presented here is effective in detecting mutations in this region in a TILLING population although false positives are detected by independent HRM analysis or Mutation Surveyor^®^. It is important to use both assays for confirming a mutation. False positives from HRM analysis may be due to the presence of some non-specific amplification, or differences in PCR amplification between samples. DNA from the TILLING population was extracted with a high-throughput method; therefore, there may be variations among samples in DNA quality, salt and inhibitor concentrations, which may affect PCR performance and HRM analysis [17]. A degree of variation in melting behavior observed within non-mutants of clinical samples was previously reported [15]. With careful DNA extraction and quantitative control, the false positive rate may be reduced to a lower level. False positives from Mutation Surveyor^® ^analysis can be controlled to a low level by using highly stringent criteria to identify mutations.

Amplicon length and sequence content may affect the sensitivity of HRM. Shorter amplicons are preferred for higher sensitivity. However, considering throughput and efficiency of TILLING, relative longer amplicons (200-250 bp) are still practical for TILLING as demonstrated in this report. False positives or negatives from HRM analysis may reduce the mutation detection accuracy. However, further sequence analysis by Mutation Surveyor^® ^will increase the accuracy. Furthermore the cost of sequencing will be largely reduced if HRM is followed by sequencing. Detecting mutations in a TILLING population is not like genotyping of medical samples, which requires 100% accuracy and sensitivity. Missing an occasional mutant will not greatly affect mutant discovery by TILLING. If deleterious mutants are identified, they can be assigned to a particular genome within bread wheat (A, B or D). This can be achieved either by cloning and sequencing the particular PCR products as shown in this report, or by using genome-specific and SNP-specific primers. Because such mutations represent a small percentage of total mutations from EMS mutagenesis, the extra work for such genome assignments should not be large.

HRM can be applied for mutation detection and SNP genotyping in medical research [21]. Application of HRM in plant research is limited. Recent publications in plants demonstrated that HRM is a useful tool for genetic variation discovery and genotyping including SNPs, INDELs and microsatellites [22-24]. To our knowledge, this is the first report of the use of HRM analysis to detect a minor sequence change in mixed PCR fragments of an EMS-treated TILLING population. Among the three different amplicons we studied in this report, HRM of ABD12-22 had the highest sensitivity for detecting mutations. ABD12-22 is the shortest (167 bp) and has the fewest intrinsic SNPs (3 SNPs) between homeoloci. The other amplicons ABD6-1 and ABD2-9 are longer (210 bp and 235 bp respectively) and more complicated (4 SNPs and 8 SNPs respectively). HRM sensitivity is determined by the sequence context, length and divergence in a PCR amplicon containing homoeologous gene fragments. HRM is usually applicable when the melting peaks are clear and distinct in non-mutant samples, which can be tested before large scale experiments, in our experience. However, the maximum fragment length and sequence divergence between homeoloci where HRM remains useful for SNP or mutation detection is unknown and further experiments are required.

HRM analysis is able to detect all single base changes, with greater sensitivity for G/A and C/T changes, and lower sensitivity for A/T and G/C changes [25]. EMS alkylates guanine bases and results in G/C to A/T transitions [26]. HRM is therefore suitable for TILLING, especially EMS-TILLING. Recent development of massively parallel sequencing instruments (Roche 454, Illumina/Solexa, and AB SOLiD) makes it possible to resequence genes of interest in a mutagenized population with relatively low cost [27,28]. However, the accessibility and affordability to these technologies still needs to be considered by many laboratories. The simplicity and low cost of HRM makes it a good choice for scanning mutations in TILLING or eco-TILLING.

## Conclusion

HRM in conjunction with sequence analysis is sensitive enough to detect a heterozygous SNP in a PCR amplicon containing three homoeologous gene fragments of wheat. Genome locations of mutations need only be determined for those are predicted to be deleterious to gene function. This method can be used for screening three homoeologous genes simultaneously, especially in a conserved functional domain or EST sequences. For diploid species, HRM scanning can be used for pooled samples. It may also be useful for SNP marker development and eco-TILLING.

## Methods

### TILLING population

An EMS TILLING population was generated in Australian wheat cultivar Ventura, and DNA samples were prepared as described previously [19].

### Test of Mutation Surveyor^® ^sensitivity

A heterozygous mutant (G1642A in *Wx-D1*) identified during screening for waxy gene mutants [19] was used to verify that Mutation Surveyor^® ^is able to detect a heterozygous mutant in a mixed DNA pool. DNA from this heterozygous mutant and a homozygous non-mutant sample were mixed to give mutant:non-mutant DNA ratios of 1:0, 1:1, 1:2, 1:3, 1:4 and 1:5. PCR was performed with these different pools using the primer set Wx7D3 [2] and the PCR products were purified with Wizard^® ^SV Gel and PCR Clean-up system (Promega, Madison, WI, USA) and Sanger-sequenced in both directions (Australia Genome Research Facility, Brisbane, Australia). Mutation Surveyor^® ^software was used for analysis of sequence data with the program set to check 2D (bi-directional) small peaks; the mutation-calling parameters were set to the program default including the overlapping factor and dropping factor. The overlapping factor is calculated by the software from the two different bases in the reference and sample traces on either side of the mutation. The dropping factor is determined from the relative intensities of the four neighboring peaks (two peaks on each side) between samples traces and reference traces. Output reports were displayed in the advanced two direction setting. In this setting the software will search for peaks buried within the baseline and indicate their presence with a short green bar if they are of the same wavelength and are in the same spatial position in both strands of sequence data.

In the analysis of *SSII *fragments, which has three homoeoloci sequence traces, certain "mutations" were deleted when the same "mutation" appeared multiple times in the same position, because they were SNPs between homoeologous loci or were due to artefacts of sequencing. 2D small peaks identified by the program were checked by examining the GAD (Graphic Analysis Display), the raw sequence chromatographs, and also using the bias of EMS mutagenesis which mutates G/C to A/T [26].

### PCR of *SSII *and HRM analysis

PCR primers used to amplify part of the carboxyl terminal domain of the *SSII *gene (GenBank accessions AB201445, AB201446 and AB201447) were designed using Primer3 version 0.4.0 http://frodo.wi.mit.edu/primer3 and manually justified to avoid regions containing SNPs among the three genes. Primers ABDF6: 5'-CCGTTCACCGAGTTGCCTG-3' and ABDR9: 5'-GGTGCTCCCGCTCGAAGTG-3' amplify a 532 bp fragment of all three homoeologous genes (Figure 2). PCR amplification was carried out in a 50 μl volume containing 2 μl of DNA (~100 ng), 1× PfuUltra^®^II buffer (Stratagene, La Jolla, CA, USA), 1× enhancer solution (Invitrogen, Carlsbad, CA, USA), 0.2 mM dNTPs, 0.25 μM primers and 1.25 U PfuUltra^®^II Fusion HS DNA Polymerase (Stratagene, La Jolla, CA, USA). PCR was conducted using a thermal cycler (MasterCycler 5333, Eppendorf, North Ryde, NSW, Australia) as follows: 95°C for 2 min, followed by 6 cycles of touchdown PCR (98°C for 10 s, an annealing step starting at 72°C for 20 s and decreasing 1°C per cycle, a temperature ramp increasing 0.5°C per second to 72°C, and 72°C for 30 min), then 35 more cycles of PCR (98°C for 10 s, 66°C for 20 s and 72°C for 15 s) and finally extension at 72°C for 1 min. PCR products were purified using Promega Wizard^® ^SV 96 PCR Clean-up kit (Promega, Madison, WI, USA) according to the manufacturer's instructions and eluted in 100 μl H_2_O. The purified PCR products were then sent to AGRF (Australia Genome Research Facility, Brisbane, Australia) for sequencing in both directions, and were used for nested PCR and HRM analysis.

Nested PCR used primers ABDF6 and ABDR1 (5'-ACGATGCCGCGGGTC-3') for a 210 bp amplicon; primers ABDF12 (5'-GGTACCTGTGGGAGCTSAAG-3') and ABDR22 (5'-CAGGGAGAAGTTGGTGTAGC-3') for a 167 bp amplicon; primers ABDF2 (5'-ACGCTGGACTCCGGCAA-3') and ABDR9 for a 235 bp amplicon; and primers ABDF3 (5'-CCTGGACGGGCAGAAGG-3') and ABDR9 for a 137 bp amplicon (Figure 2). PCR was performed in 10 μl reactions under the same conditions as above, except that 2.5 μM CYTO^®^9 (Invitrogen, Carlsbad, CA, USA) was added to the reactions and 1 μl of a 100× dilution of the first PCR (unpurified) or 1 μl of 20× dilution of purified first PCR product was used as the template. PCR and HRM analysis were carried out in a Rotor-Gene™ 6000 real time PCR machine (Corbett Research, Mortlake, NSW, Australia) set at the following conditions: 1 cycle of 95°C for 3 min; 40 cycles of 95°C for 10 s, 60°C for 15 s, 72°C for 10 s; 1 cycle of 72°C for 90 s and a melt from 72°C to 90°C rising at 0.1°C per step (wait 2 s every step). The amplification was monitored. Significantly early or late amplifications were omitted in HRM analysis, as they may give rise to aberrant melting curves. After the PCR and melting steps, samples were loaded on 2% agarose gels to check whether amplifications were specific.

### Cloning

PCR products of mutant samples were cloned into the pGEM^®^-T Easy vector (Promega, Madison, WI, USA) according to the manufacturer's instructions. Clones were sequenced to identify the genome locations of mutations.

## Authors' contributions

KV performed mutagenesis and DNA sample preparation. CD designed experiments, performed HRM and Mutation Surveyor^® ^analysis, cloning, and wrote the paper. PS participated in its design and coordination and helped to draft the manuscript. All authors read and approved the final manuscript.

## Supplementary Material

Additional file 1 **Initial analysis with Mutation Surveyor^® ^in ABD6-9 sequence traces identified 26 mutants in 192 TILLING lines**. Initial analysis with Mutation Surveyor^® ^in ABD6-9 sequence traces identified 26 mutants in 192 TILLING lines. Click here for file

Additional file 2 **Amplicon melting analysis of fragment ABD12-22**. Amplicon melting analysis of fragment ABD12-22 in duplicated non-mutant and mutant samples, showing normalized melting curve (A), difference plot (B) and derivative melting curve (C). Non-mutants are shown in red and black (thick lines). Mutants (as in Additional file 1) are M9 (G166A, green), M10 (C169T, blue), M11 (C177T, orange) and M13 (C205T, pink). Click here for file

Additional file 3 **Amplicon melting analysis of fragment ABD2-9**. Amplicon melting analysis of fragment ABD2-9 in duplicated non-mutant and mutant samples, showing normalized melting curve (A), difference plot (B) and derivative melting curve (C). Non-mutants are shown in red and black (thick lines). Mutants (as in Additional file 1) are M18 (G348A, blue), M19 (C360T, brown), M20 (C366T, pink), M21 (C379T, green), M22 (G383A, orange), M24 (G462A, purple), and M25 (C463T and C489T, aqua). Click here for file
