# Supplementary material for: Simultaneous mutation detection of three homoeologous genes in wheat by High Resolution Melting analysis and Mutation Surveyor®
Source: BMC Plant Biol. 2009 Dec 4;9:143. doi: 10.1186/1471-2229-9-143 (PMC2794869; doi:10.1186/1471-2229-9-143)
Supplement: Additional file 1 — Initial analysis with Mutation Surveyor® in ABD6-9 sequence traces identified 26 mutants in 192 TILLING lines. Initial analysis with Mutation Surveyor® in ABD6-9 sequence traces identified 26 mutants in 192 TILLING lines. [file 1471-2229-9-143-S1.DOC]

### Additional file 1: Initial analysis with Mutation Surveyor® in ABD6-9 sequence traces identified 26 mutants in 192 TILLING lines.

| **No** | **Sample** | **Mutation Surveyor report** | **Position in**  **ABD6-9** | **Position in Gene (SSII-A)** | **Codon change** | **Amino acid change** |
| --- | --- | --- | --- | --- | --- | --- |
| M1 | 3D7 | (16)C>CT$7 | C34T | C5999T | cac/tac | H501Y |
| M2 | 1D3 | (25)C>CT$10 | C43T | C6008T | ctg/ttg | L504L |
| M3^1^ | 3H5 | (40)G>AG$7 | G58A | G6023A | ggt/agt | G509S |
| M4^2^ | 3F10 | (65)C>CT$7 | C83T | C6048T | gcc/gtc | A517V |
| M5^2^ | 1C8 | (85)G>AG$7 | G103A | G6068A | gac/aac | D524N |
| M6^2^ | 1F10 | (115)C>CT$7 | C133T | C6098T | ctg/ttg | L534L |
| M7^2^ | 1D8 | (129)G>AG$7 | G147A | G6112A | aag/aaa | K538K |
| M8 | 3E5 | (129)G>AG$7 | G147A | G6112A | aag/aaa | K538K |
| M9^2^ | 3A8 | (148)G>AG$7 | G166A | G6131A | ggg/agg | G545R |
| M10 | 1D9 | (151)C>CT$28 | C169T | C6134T | ctt/ttt | L546F |
| M11 | 3A4 | (159)C>CT$11 | C177T | C6142T | gac/gat | D548D |
| M12^1^ | 3F3 | (167)G>AG$7 | G185A | G6150A | cgg/cag | R551Q |
| M13 | 3H9 | (187)C>CT$7 | C205T | C6170T | cgc/tgc | R558C |
| M14^1^ | 1F7 | (272)C>CT$7 | C290T | C6255T | tcc/ttc | S586F |
| M15^1^ | 3A12 | (295)A>AC$7 | A313C | A6278C | aag-cag | K594Q |
| M16 | 1D5 | (300)G>AG$7 | G318A | G6283A | cgg/cga | R595R |
| M17 | 3D8 | (306)C>CT$7 | C324T | C6289T | tgc/tgt | C597C |
| M18^1^ | 1A1 | (330)G>AG$7 | G348A | G6313A | ctg/cta | L605L |
| M19 | 3F6 | (342)C>CT$7 | C360T | C6325T | gtc/gtt | V609V |
| M20^1^ | 3G3 | (348)C>CT$7 | C366T | C6331T | gcc/gct | A611A |
| M21 | 1B2 | (361)C>CT$13 | C379T | C6344T | ctc/ttc | L616F |
| M22 | 3B7 | (365)G>AG$7 | G383A | G6348A | ggc/gac | G617D |
| M23 | 1E4 | (387)G>AG$7 | G405A | G6370A | ggg/gga | G624G |
| M24^1^ | 1G1 | (444)G>AG$7 | G462A | G6427A | gtg/gta | V643V |
| M25^1^ | 3D9 | (445)C>CT$7 | C463T | C6428T | cag/tag | Q644Stop |
| M26^1^ | 3D9 | (471)C>CT$7 | C489T | C6454T | cgc/cgt | R652R |

## ^1^These samples were identified by HRM analysis and re-analysis of sequences as false positives.

## ^2^These samples were detected as mutants in the HRM analysis of 32 samples.
