# Supplementary figures and images for: Simultaneous mutation detection of three homoeologous genes in wheat by High Resolution Melting analysis and Mutation Surveyor®
Source: BMC Plant Biol. 2009 Dec 4;9:143. doi: 10.1186/1471-2229-9-143 (PMC2794869; doi:10.1186/1471-2229-9-143)

**A**


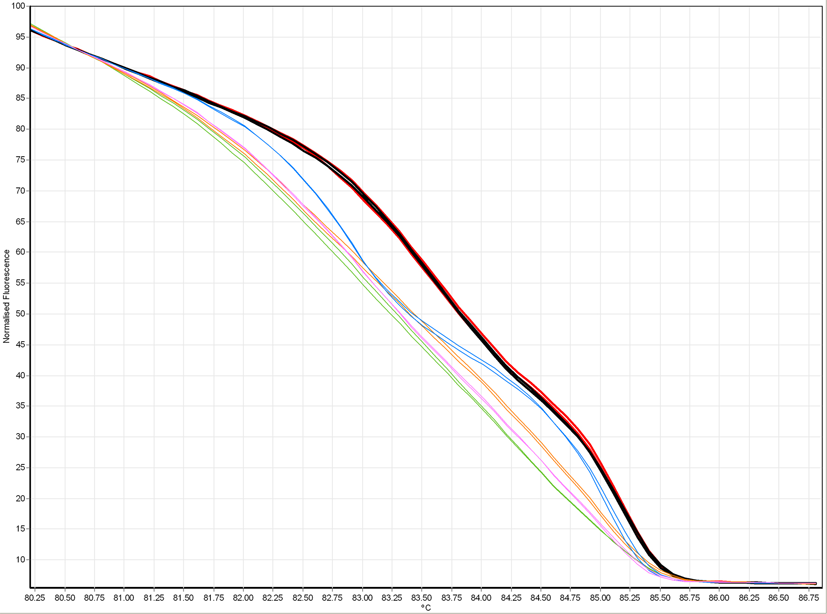


Normalized Fluorescence

**B**


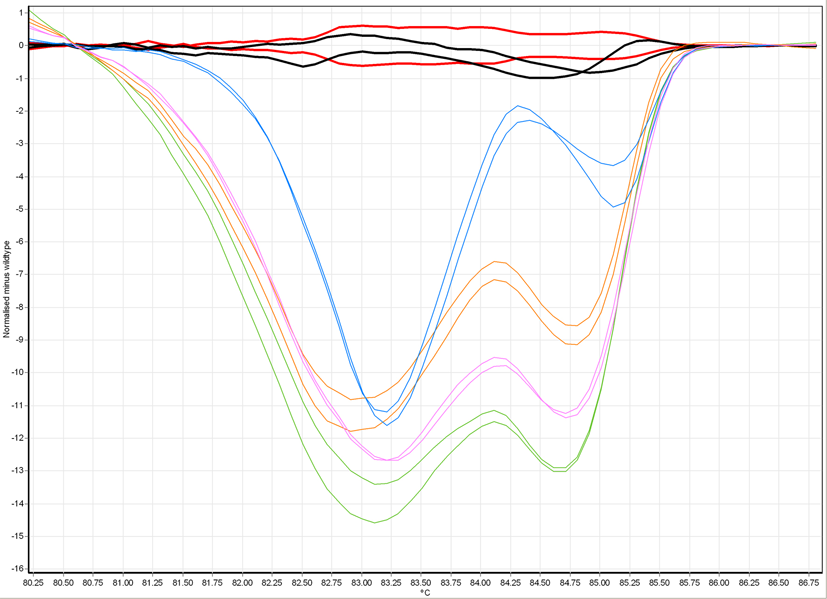


Relative Signal Difference

**C**


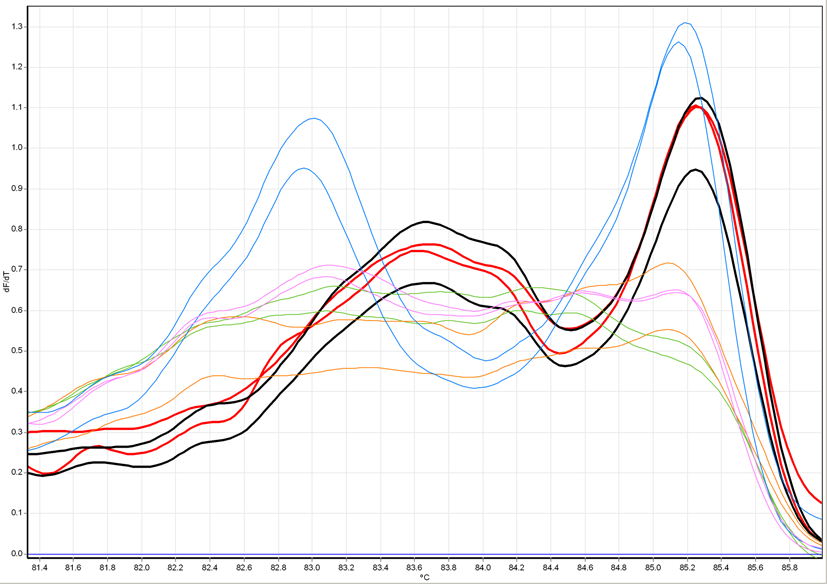


-dF/dT

Temperature (˚C)

Supplement: Additional file 2 — Amplicon melting analysis of fragment ABD12-22. Amplicon melting analysis of fragment ABD12-22 in duplicated non-mutant and mutant samples, showing normalized melting curve (A), difference plot (B) and derivative melting curve (C). Non-mutants are shown in red and black (thick lines). Mutants (as in Additional file 1) are M9 (G166A, green), M10 (C169T, blue), M11 (C177T, orange) and M13 (C205T, pink). [file 1471-2229-9-143-S2.DOC]

**A**


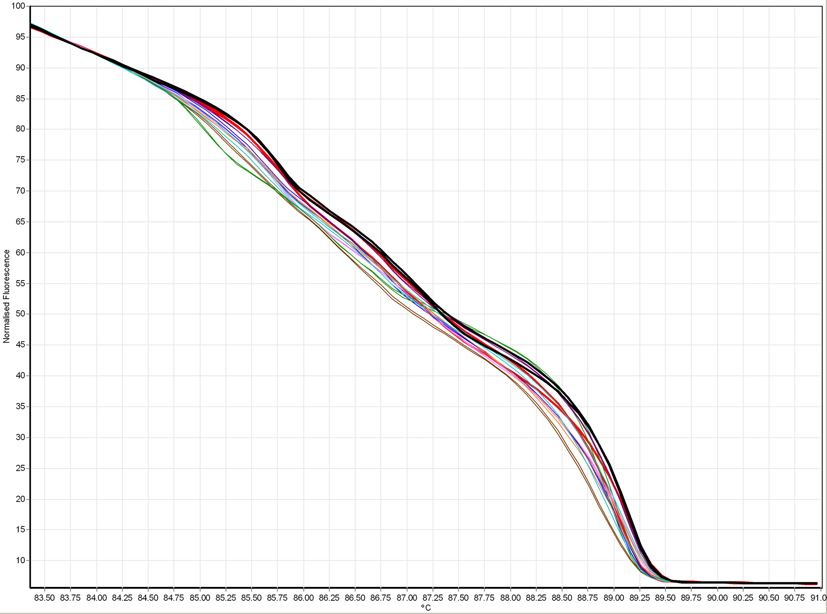


Normalized Fluorescence

**B**


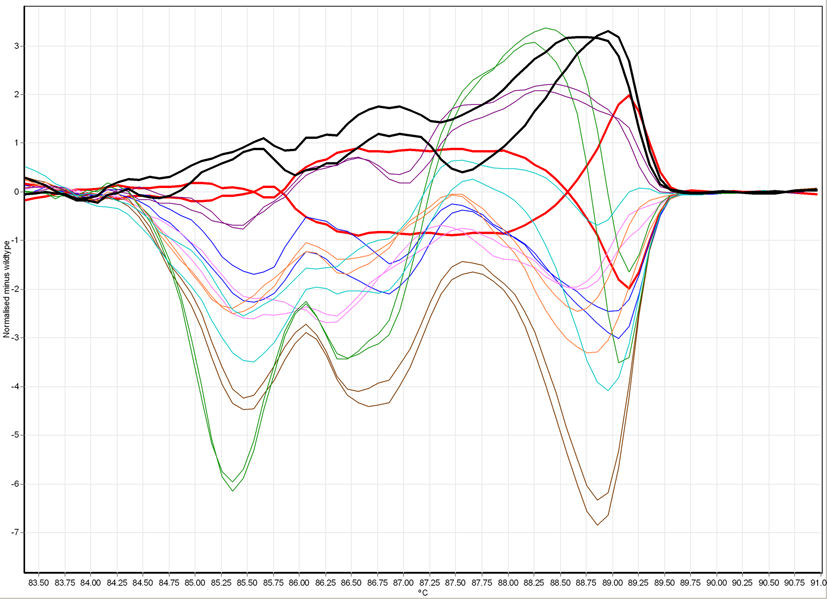


Relative Signal Difference

**C**


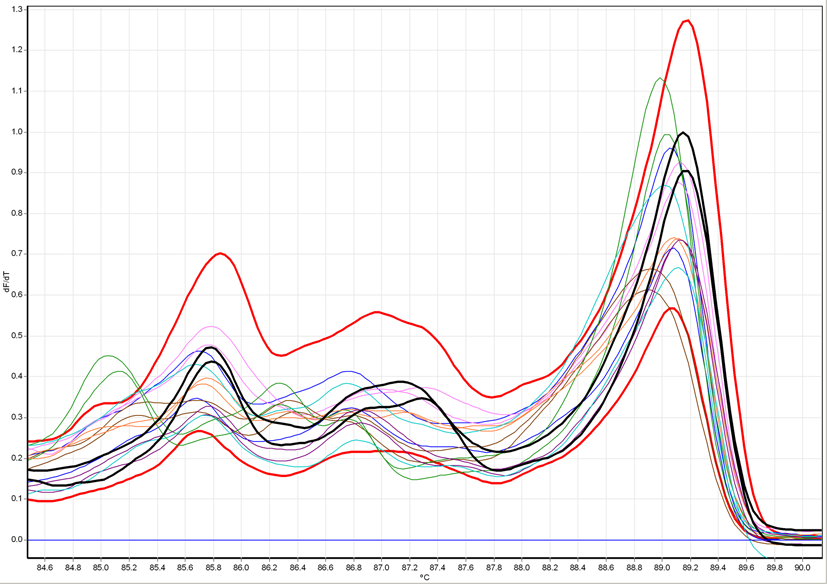


-dF/dT

Temperature (˚C)

Supplement: Additional file 3 — Amplicon melting analysis of fragment ABD2-9. Amplicon melting analysis of fragment ABD2-9 in duplicated non-mutant and mutant samples, showing normalized melting curve (A), difference plot (B) and derivative melting curve (C). Non-mutants are shown in red and black (thick lines). Mutants (as in Additional file 1) are M18 (G348A, blue), M19 (C360T, brown), M20 (C366T, pink), M21 (C379T, green), M22 (G383A, orange), M24 (G462A, purple), and M25 (C463T and C489T, aqua). [file 1471-2229-9-143-S3.DOC]
